# Supplementary material for: A Novel Preanalytical Strategy Enabling Application of a Colorimetric Nanoaptasensor for On-Site Detection of AFB1 in Cattle Feed
Source: Sensors (Basel). 2022 Nov 29;22(23):9280. doi: 10.3390/s22239280 (PMC9735511; doi:10.3390/s22239280)
Supplement: Supplementary file 1 [file sensors-22-09280-s001.zip › sensors-1992017-supplementary.pdf]

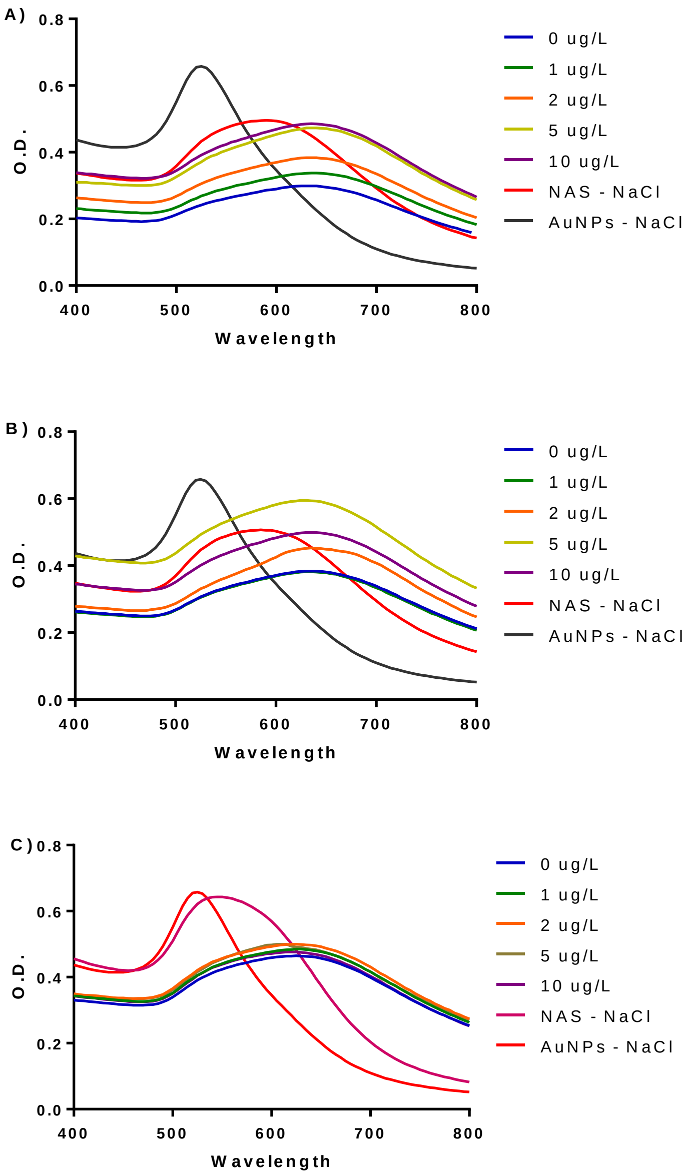

Figure S1. Spectral variation associated with AuNPs aggregation of nanoaptasensors detecting AFB1 with 1:10 AuNPs aptamer molar ratio (A), 1:20 AuNPs aptamer molar ratio (B), and 1:40 AuNPs aptamer molar ratio (C), upon NaCl addition. The results were averaged from independent experiments for each of nanoaptasensor (n=3).

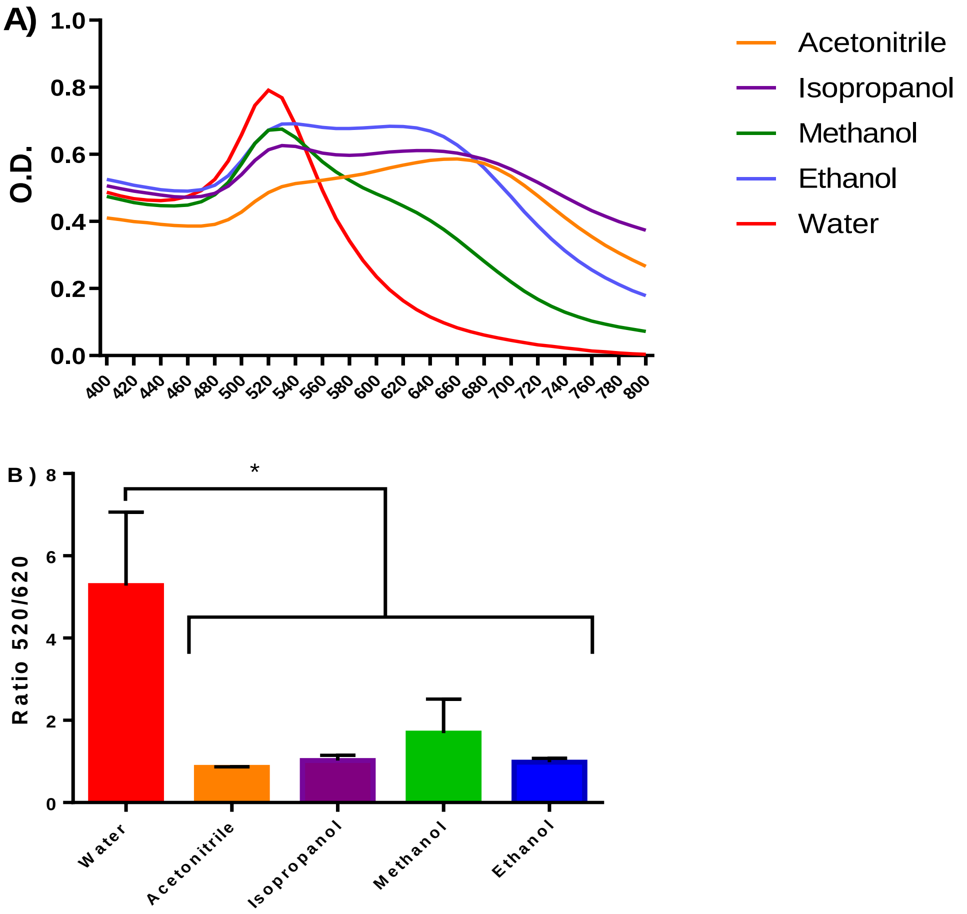

Figure S2. AuNPs aggregation in different solvents. A) Absorption spectrum resulting from addition to acetonitrile, isopropanol, methanol, ethanol, and water of dispersed AuNPs. B) Spectral changes upon aggregation of AuNPs in different solvents (A_{520}/A_{620}). Results were averaged from three independent experiments (n=3). Data were analyzed using the nonparametric Mann–Whitney test. Statistically significant differences compared with the controls and different treatments are indicated. * = $p< 0.05$.
